# Supplementary material for: Intra-Articular Injections of Autologous Adipose Tissue or Platelet-Rich Plasma Comparably Improve Clinical and Functional Outcomes in Patients with Knee Osteoarthritis
Source: Biomedicines. 2022 Mar 16;10(3):684. doi: 10.3390/biomedicines10030684 (PMC8945733; doi:10.3390/biomedicines10030684)
Supplement: Supplementary file 1 [file biomedicines-10-00684-s001.zip › biomedicines-1593250-supplementary.pdf]

Supplementary material

Article

Intra-articular injections of autologous adipose tissue or platelet-rich plasma comparably improve clinical and functional outcomes in patients with knee osteoarthritis

Jakub Kaszyński[1#], Paweł Bąkowski[1*#], Bartosz Kiedrowski[1], Łukasz Stołowski[1], Anna Wasilewska-Burczyk[2], Kamilla Grzywacz[2] and Tomasz Piontek[1, 3]

1 Department of Orthopedic Surgery, Rehasport Clinic, Poznań, Poland; jakub.kaszynski@rehasport.pl (J.K.); pawel.bakowski@rehasport.pl (P.B.); bartosz.kiedrowski@rehasport.pl (B.K.); lukasz.stolowski@rehasport.pl (Ł.S.)
2 Institute of Bioorganic Chemistry Polish Academy of Sciences, Poznań, Poland; awasilewska@ibch.poznan.pl (A.W.-B.); kgrzywacz@ibch.poznan.pl (K.G.)
3 Department of Spine Disorders and Pediatric Orthopedics, University of Medical Sciences Poznań, Poznań, Poland
* Correspondence: pawel.bakowski@rehasport.pl
# Co-first authorship statement: Paweł Bąkowski and Jakub Kaszyński have contributed equally to the work.

Table S1: The sequences of the primers used in this study.

GENE	Forward	Reverse
IL23A	ACACATGGATCTAAGAGAAGAGGG	AGAGAAGGCTCCCCTGTGAA
IL1B	CTCAAGTGATCCACCCACCT	CTCAAGTGATCCACCCACCT
CXCL1	CCAGCTCTTCCGCTCCTC	CACGGACGCTCCTGCTG
CXCL3	AGATACTGAACAAGGGGAGCAC	CCTTTCCAGCTGTCCCTAGA
CCL5	GAGGCTTCCCCTCACTATCC	CTCAAGTGATCCACCCACCT
MMP3	CCCATATATGCCTGCTGTCC	ACAGGTTGATTCCTGGTCA
GAPDH	ATCAAGAAGGTGGTGAAGCA	GTCGCTGTTGAAGTCAGAGGA

Table S2: Patient demographic.

	PRP group	AAT group	Control group
Age [y.o.]	57 ± 8 (45-69)	55 ± 8 (43-67)	56 ± 9 (45-73)
BMI [kg/mc^2]	26 ± 3 (19-31)	27 ± 3 (22-37)	26 ± 2 (18-33)
Limb [n]	20	20	20
dominant	12	12	n.d.
nondominant	8	8	n.d.
KL grade [n]	20	20	20
I	0	0	n.d.
II	12	11	n.d.
III	8	9	n.d.
IV	0	0	n.d.

Table S3: The results of the VAS, WOMAC, IKDC 2000 and EQ-5D-5L evaluation.

	**Pretreatment**	**1M FU**	**3M FU**	**6M FU**	**12M FU**
**VAS**					
PRP group	5.2 ± 1.9 (1-8)	4.1 ± 2.7 (0-8)	3.2 ± 2.4 (0-7)	3.2 ± 2.1 (0-8)	3.0 ± 2.2 (0-9)
AAT group	5.1 ± 2.5 (0-8)	4.1 ± 1.9 (1-8)	3.7 ± 2.5 (1-10)	3.1 ± 2.2 (0-8)	2.8 ± 2.2 (0-8)
Control group	0.1 ± 0.4 (0-2)	n.d.	n.d.	n.d.	n.d.
**WOMAC**					
PRP group	70.1 ± 20.6 (20.8-95.8)	77.6 ± 19.5 (31.2-100.0)	79.1 ± 20.1 (35.4-100.0)	83.2 ± 14.9 (42.7-100.0)	83.8 ± 17.3 (28.1-100.0)
AAT group	63.9 ± 16.2 (35.4-90.6)	78 ± 13.6 (50.0-99.0)	79 ± 11.6 (50.0-99.1)	78.9 ± 14.3 (47.9-100.0)	83.6 ± 11 (67.7-100.0)
Control group	98.2 ± 2.0 (94.8-100.0)	n.d	n.d	n.d	n.d
**IKDC 2000**					
PRP group	49.5 ± 19.5 (18.2-84.4)	57.9 ± 20 (26-93.5)	62.7 ± 21.6 (23.4-100.0)	64.3 ± 18.8 (26.0-97.4)	66.3 ± 19.7 (24.7-94.8)
AAT group	44.2 ± 13.1 (15.6-68.8)	51.2 ± 11.6 (26.0-72.7)	60.3 ± 12.6 (29.8-81.8)	63.3 ± 14.6 (35.1-89.6)	68.9 ± 15.95 (41.6-100.0)
Control group	90.5 ± 6.8 (76.6-100.0)	n.d	n.d	n.d	n.d
**EQ-5D descriptive system**					
PRP group	66.5 ± 17.6 (30.0-95.0)	73.8 ± 13.1 (50.0-96.0)	75.9 ± 14.2 (45.0-100.0)	78.2 ± 13.6 (40.0-100.0)	78.7 ± 12.1 (50.0-100.0)
AAT group	66.5 ± 17.6 (30.0-95.0)	70.9 ± 16.8 (20.0-96.0)	72.3 ± 17.0 (20.0-95.0)	76.4 ± 13.7 (45.0-100.0)	80.2 ± 12.4 (50.0-100.0)
Control group	76.9 ± 19.0 (45.0-100.0)	n.d	n.d	n.d	n.d
**EQ visual analogue scale**					
PRP group	0.7 ± 0.2 (0.1-1.0)	0.7 ± 0.1 (0.5-0.8)	0.7 ± 0.1 (0.5-1.0)	0.8 ± 0.1 (0.6-1.0)	0.8 ± 0.2 (0.6-1.0)
AAT group	0.7 ± 0.2 (0.1-1.0)	0.7 ± 0.3 (0.1-1.0)	0.8 ± 0.2 (0.1-1.0)	0.8 ± 0.1 (0.3-1.0)	0.8 ± 0.2 (0.2-1.0)
Control group	0.9 ± 0.1 (0.8-1.0)	n.d	n.d	n.d	n.d

Mean ± standard deviation, minimum and maximum value is presented. 1M FU – 1 month follow-up, 3M FU – 3 months follow-up, 6M FU – 6 months follow-up, 12M FU – 12 months follow-up, n.d. – not defined.

Table S4: The results of the KOOS evaluation.

	Pretreatment	1M FU	3M FU	6M FU	12M FU
**KOOS Pain**					
PRP group	65.3 ± 20.2 (25.0-94.9)	73.8 ± 19.5 (33.3-100.0)	76.2 ± 21.8 (27.8-100.0)	79.3 ± 17.2 (31.3-100.0)	81.5 ± 17.1 (25.0-100.0)
AAT group	57.8 ± 15.8 (33.3-88.9)	75.2 ± 11.5 (55.6-94.4)	75.8 ± 12.6 (55.6-94.4)	75.1 ± 13.2 (44.4-100.0)	78.9 ± 13.2 (55.6-100.0)
Control group	99.4 ± 1.1 (97.2-100.0)	n.d	n.d	n.d	n.d
**KOOS Other Symptoms**					
PRP group	66.6 ± 18.2 (32.1-96.4)	70.3 ± 21.7 (17.9-100.0)	76.3 ± 19.7 (28.6-100.0)	75.7±16.9 (28.6-100)	79.3±16.3 (28.6-100)
AAT group	57.7 ± 17.6 (28.6-89.3)	74.8 ± 13.9 (57.1-100.0)	75.0 ± 11.8 (57.1-100.0)	75.4 ± 16.2 (42.9-100.0)	78.9 ± 16.9 (39.3-100.0)
Control group	97.1 ± 3.2 (92.9-100.0)	n.d	n.d	n.d	n.d
**KOOS Function in Daily Living**					
PRP group	70.1 ± 21.5 (19.1-97.1)	78.1 ± 19.2 (32.4-100.0)	78.7 ± 20.3 (35.3-100.0)	83.4 ± 15.0 (45.6-100.0)	83.6 ± 18.0 (30.9-100.0)
AAT group	63.7 ± 17.0 (33.8-91.2)	78.4±15.3 (42.6-100.0)	79.1±11.3 (42.6-100.0)	79.3 ± 15.8 (45.6-100.0)	84.0±11.6 (63.3-100.0)
Control group	98.0 ± 2.3 (94.1-100.0)	n.d	n.d	n.d	n.d
**KOOS Function in Sport and Recreation**					
PRP group	42.4 ± 23.7 (0.0-75.0)	54.1 ± 26.4 (10.0-90.0)	55.8 ± 30.4 (0.0-100.0)	60.8 ± 25.3 (0.0-95.0)	61.5 ± 28.8 (0.0-100.0)
AAT group	35.5 ± 24.6 (0.0-90.0)	51.2 ± 22.2 (0.0-100.0)	53.1 ± 18.2 (0.0-100.0)	54.8 ± 23.7 (0.0-100.0)	66.1 ± 21.2 (20.0-100.0)
Control group	90.3±9.1 (75-100)	n.d	n.d	n.d	n.d
**KOOS knee-related Quality of Life**					
PRP group	42.5 ± 14.4 (18.8-62.5)	51.3 ± 20.2 (25.0-93.8)	57.8 ± 23.5 (12.5-100.0)	54.1 ± 18.6 (25.0-93.8)	59. 2± 20.5 (12.5-100.0)
AAT group	38.8 ± 7.2 (31.3-56.3)	50.3 ± 13.1 (31.3-87.5)	51.3 ± 9.1 (31.8-89.5)	55.9 ± 17.0 (31.3-100.0)	62.2 ± 18.2 (37.5-100.0)
Control group	100±0 (100-100)	n.d	n.d	n.d	n.d
**KOOS Index**					
PRP group	62.6 ± 19.4 (21.4-89.0)	70.4 ± 19.3 (26.8-96.4)	73.0 ± 20.7 (29.8-100.0)	75.7 ± 16.0 (34.8-97.0)	77.4 ± 17.8 (28.0-100.0)
AAT group	55.7 ± 14.7 (29.2-79.2)	71.1 ± 12.4 (50.6-97.1)	71.1 ± 10.4 (50.8-99.2)	72.5 ± 14.3 (39.9-100.0)	77.9 ± 13.1 (58.3-100.0)
Control group	97.4 ± 2.5 (93.5-100.0)	n.d	n.d	n.d	n.d

Mean ± standard deviation, minimum and maximum value is presented. 1M FU – 1 month follow-up, 3M FU – 3 months follow-up, 6M FU – 6 months follow-up, 12M FU – 12 months follow-up, n.d. – not defined.

Table S5: The results of the functional evaluation.

	**Pretreatment**	**1M FU**	**3M FU**	**6M FU**	**12M FU**
**TUG**					
PRP group	5.8 ± 1.1 (3.9-7.6)	5.4 ± 0.9 (3.4-6.6)	5.1 ± 0.7 (3.5-6.4)	5.0 ± 0.6 (3.8-6.0)	5.2 ± 0.7 (3.9-7.0)
AAT group	6.9 ± 1.6 (4.1-10.1)	5.9 ± 1.3 (3.7-8.0)	5.7 ± 1.1 (3.9-7.3)	5.3 ± 1.0 (3.6-6.6)	5.4 ± 0.9 (3.9-6.8)
Control group	5.2 ± 0.8 (4.2-7.0)	n.d	n.d	n.d	n.d
**5xSTS**					
PRP group	9.3 ± 2.1 (5.5-14.7)	8.0 ± 1.7 (4.5-11.8)	7.6 ± 1.6 (4.3-10.5)	7.6 ± 1.5 (4.5-11.4)	7.6 ± 1.7 (4.8-12.0)
AAT group	11.1 ± 2.7 (6.1-15.2)	9.9 ± 2.2 (5.7-13.4)	8.7 ± 1,8 (5.8-11.7)	8.3 ± 2.0 (5.1-12.0)	8.3 ± 1.9 (5.4-12.1)
Control group	7.1 ± 1.5 (4.3-9.7)	n.d	n.d	n.d	n.d
**10mWT**					
PRP group	5.3 ± 0.9 (3.2-6.8)	4.9 ± 0.8 (2.8-6.1)	4.9 ± 0.7 (3.2-5.9)	4.7 ± 0.7 (3.1-5.7)	4.8± 0.7 (3.4-6.8)
AAT group	6.2 ± 1.6 (3.6-9.1)	5.4 ± 1.3 (3.2-7.6)	5.1 ± 1.2 (3.3-6.8)	4.9 ± 1.1 (3.2-6.5)	4.8 ± 0.9 (3.2-6.4)
Control group	4.6 ± 0.8 (3.1-6.1)	n.d	n.d	n.d	n.d

Mean ± standard deviation, minimum and maximum value is presented. 1M FU – 1 month follow-up, 3M FU – 3 months follow-up, 6M FU – 6 months follow-up, 12M FU – 12 months follow-up, n.d. – not defined.

Table S6: The results of the isokinetic evaluation.

	**Pretreatment**	**1M FU**	**3M FU**	**6M FU**	**12M FU**
**MVIC extensors**					
PRP group	3.3 ± 1.3 (1.9-6.1)	3.6 ± 1.3 (2.1-6.8)	3.7 ± 1.4 (1.4-7.2)	3.9 ± 1.3 (1.8-6.9)	3.9 ± 1.4 (2.0-8.2)
AAT group	3.3 ± 1.5 (0.9-7.1)	3.5 ± 1.1 (1.8-6.5)	4.1 ± 1.2 (2.5-7.0)	4.2 ± 1.3 (1.7-7.0)	4.2 ± 1.1 (2.2-5.6)
Control group	4.3 ± 1.3 (2.5-6.8)	n.d	n.d	n.d	n.d
**MVIC flexors**					
PRP group	1.7 ± 0.5 (0.9-2.9)	1.8 ± 0.6 (0.9-3.4)	1.8 ± 0.5 (1.0-3.1)	2.0 ± 0.6 (0.9-3.3)	1.8 ± 0.6 (0.9-3.0)
AAT group	2.0 ± 0.6 (1.0-3.0)	2.0 ± 0.7 (0.9-3.2)	2.2 ± 0.6 (0.9-3.4)	2.1 ± 0.6 (0.9-3.2)	2.2 ± 0.6 (1.2-3.2)
Control group	1.9 ± 0.6 (1.1-3.2)	n.d	n.d	n.d	n.d

Mean ± standard deviation, minimum and maximum value is presented. 1M FU – 1 month follow-up, 3M FU – 3 months follow-up, 6M FU – 6 months follow-up, 12M FU – 12 months follow-up, n.d. – not defined.
